# The Sir Andrew Clark Memorial

**Published:** 1895-10-12

**Authors:** W. E. Gladstone


### THE SIR ANDREW CLARK MEMORIAL.

The editor of the *Weekly Sun* has received the following letter from Mr. Gladstone:—

Dear Sir,—I deeply regret the failure of the subscription for the Clark Memorial. Were I called upon to advise about it I should be much tempted to recommend that an application for a small subscription be made to every medical man in Great Britain. I know not whether it is now too late; but I think the main interest in the design is that of the great profession with which Sir A. Clark was identified in feeling and in history.—I remain, dear Sir, your very faithful, W. E. Gladstone.

Mr. Gladstone can not have been aware of the terms of the original circular or advertisement upon which subscriptions were invited. We think it may be useful to reprint this advertisement (*Times*, Feb. 10th, 1894), which men of business must agree makes it essential that the London Hospital Committee shall either return all the money subscribed, without any deduction for expenses, or erect the memorial originally proposed at a cost of £12,000.

Memorial to the late Sir Andrew Clark, Bart., M.D.—At a meeting held at the Horse Guards under the chairmanship of H.R.H. the Duke of Cambridge, K.G., on January 11th, it was unanimously resolved to raise a fund with the object of establishing a memorial to the late Sir Andrew Clark, Bart., M.D. It was determined that an effort should be made to raise sufficient money to build new isolation wards and a new pathological department at the London Hospital, to be called the "Andrew Clark Memorial Buildings." *Sir Andrew Clark first came to London in* 1853 *to join the London Hospital. From that time to the hour of his death he was enthusiastically devoted to its service.* It was there he gained the wide experience which has proved so valuable to all classes of the community. It is earnestly hoped that all friends of the late distinguished physician, including those who have benefited by his professional skill, will assist the committee of this fund in their efforts to do honour to his memory. *The estimated cost of the work is* £12,000. *The present isolation buildings are quite inadequate. Their replacement is of urgent necessity.* It is not anticipated that the number of beds in the hospital or the cost of maintenance will be materially increased, as it is hoped that by securing greater efficiency certain economies may be effected. Due notice will be given of a public meeting to be held at an early date, at which H.R.H. the Duke of Cambridge has promised to preside, and the Right Hon. W. E. Gladstone, M.P., has expressed a hope that he may be able to speak. Subscriptions to this fund will be most thankfully received by the treasurer, Mr. J. H. Buxton, or by the hon. secretary, Mr. G. Q. Roberts, at the hospital; also by the bankers, Messrs. Robarts, Lubbock, and Co., or by Messrs. Barclay, Bevan, and Co.

